# What are the optimal cut-off points of anthropometric indices for prediction of overweight and obesity? Predictive validity of waist circumference, waist-to-hip and waist-to-height ratios

**DOI:** 10.34172/hpp.2020.23

**Published:** 2020-03-30

**Authors:** Helda Tutunchi, Mehrangiz Ebrahimi-Mameghani, Alireza Ostadrahimi, Mohammad Asghari-Jafarabadi

**Affiliations:** ^1^Student Research Committee, Nutrition Research Center, School of Nutrition & Food Sciences, Tabriz University of Medical Sciences, Tabriz, Iran; ^2^Social Determinant of Health Research Center, Tabriz University of Medical Sciences, Tabriz, Iran; ^3^Nutrition Research Center, Department of Clinical Nutrition, Tabriz University of Medical Sciences, Tabriz, Iran; ^4^Road Traffic Injury Research Center, Tabriz University of Medical Sciences, Tabriz, Iran

**Keywords:** Anthropometric indices, Iranian adults, Obesity, Receiver operating characteristics

## Abstract

**Background:** Planning for obesity prevention is an important global health priority. Our aim in this study was to find the optimal cut-off points of waist circumference (WC), waist- to- hipratio (WHR) and waist- to- height ratio (WHtR), as three anthropometric indices, for prediction of overweight and obesity. We also aimed to compare the predictive ability of these indices to introduce the best choice.

**Methods:** In this cross-sectional study, a total of 500 subjects were investigated. Anthropometric indicators were measured using a standard protocol. We considered body mass index (BMI) as the simple and most commonly used index for measuring general obesity as the comparison indicator in the present study to assess the diagnostic value for other reported obesity indices.We also performed receiver operating characteristic (ROC) curve analysis to define the optimal cut-off points of the anthropometric indicators and the best indices for overweight and obesity.

**Results:** The proposed optimal cut-offs for WC, WHtR, and WHR were 84 cm, 0.48 and 0.78 for women and 98 cm, 0.56 and 0.87 for men, respectively. The area under the ROC curve ofWHtR (women: AUC=0.97, 95% CI: 0.96-0.99 vs. men: AUC=0.97, 95%CI: 0.96-0.99) and WC(women: AUC=0.97, 95% CI, 0.95-0.99 vs. men: AUC=0.98, 95% CI: 0.97-0.99) were greater than WHR (women: AUC=0.79, 95% CI =0.74-0.85 vs. men: AUC=0.84, 95% CI=0.79-0.88).

**Conclusion:** This study demonstrated that the WC and WHtR indicators are stronger indicators compared to the others. However, further studies using desirable and also local cutoffs against more accurate techniques for body fat measurement such as computerized tumor (CT) scans and dual-energy x-ray absorptiometry (DEXA) are required.

## Introduction

The high prevalence of overweight and obesity represents the main challenge for prevention of chronic diseases.^1-5^ The first step in community health planning is screening and identifying overweight and obesity via easy and precise methods.^6^ Obesity, which is defined to an excess of body fat, can be detected accurately using dual-energy x-ray absorptiometry (DEXA) and magnetic resonance imaging (MRI) techniques.^7^ However, these methods, in addition to being expensive and time consuming, require measurement skills and they cannot be easily performed across a large population.^8^ Body mass index (BMI) is recommended as a globally accepted index for screening general obesity and can represent overall obesity without providing any information about body fat distribution and in particular, abdominal obesity as well as other limitations.^9-12^ This indicator is also the simplest and the most widely used indicator for measuring obesity in the community, and numerous studies have used it for assessing diagnostic value of other obesity indicators in terms of determining overall body fat.^10^ For example, Taylor et al demonstrated that BMI indicator was able to correctly identify 83% of people who had a higher body fat percentage using a DEXA scan.^13^ Moreover, the correlation coefficient between BMI and total body fat obtained using DEXA was very high [BMI and total body fat (kg), r = 0.91; BMI and total body fat (%), r = 0.84].^13^ Furthermore, in study performed by Moy and Atiya. ROC curves were applied to evaluate the WC and WHR values, as screening measures beside BMI (as the reference test).^14^

Several studies have suggested that waist circumference (WC) can be applied as a screening instrument for determining abdominal obesity and overweight rather than BMI.^15-17^ The measurement of WC, in addition to being less time-consuming than BMI, is a convenient and simple method.^18^ It has also been suggested that WHR can be used for assessing central obesity, visceral fat and the risk factors for chronic diseases.^15^ Also, waist circumference-to-height ratio (WHtR) is suggested as an anthropometric indicator to assess central adiposity. This index is closely related to metabolic risk factors and mortality, independent of body weight.^19,20^ Body composition differs among all ethnic groups and different populations worldwide. Thus, what is the best way to assess the obesity associated with metabolic diseases using anthropometric indices is still controversial. Moreover, the cut-off points of the anthropometric indices when used to determine overweight and obesity are based on European and American populations, which might be different when used among Middle Eastern populations (e.g., Iranian adults). Accordingly, this study aimed to find the optimal cut-off points of the three anthropometric indices including WC, WHR and WHtR for predicting overweight and obesity in a sample of Iranian adults and to compare the predictive ability of the indices to introduce the best one.

## Materials and Methods

### Study population

This conducted cross-sectional study on 500 subjects selected through simple random sampling in the northwest of Iran. According to the latest studies, the prevalence rate of obesity in populations above the age of 18 is reported to be 22% in Iran.^21^ By considering the prevalence of obesity, a sensitivity of 96% and a specificity of 60% for WC, the best indicators of abdominal obesity, a confidence interval (CI) of 95% and a power of 90%, the minimum sample size was calculated to be at least 455 subjects. However, a large sample size (n = 550) was considered to make the results more reliable. Although there were regular follow-ups and the regular encouragement of individuals to participate in the study, the rate of participation was 90%. Five hundred subjects, including 285 males and 215 females (18+ years), were eventually recruited. The setting of the study was a large industrial company in the northwest of Iran that was considered to provide a good representation of the socioeconomic characteristics, lifestyle, general health situation and crowd- structure of the population of Tabriz. Inclusion criteria included having the age between 18 and 60 years old, and BMI >25 kg/m^2^. Individuals with kidney diseases, liver and heart failure, and gastrointestinal disorders were excluded from the study. Moreover, participants who were pregnant or constantly taking medication, and those with severe diseases were excluded. A questionnaire and informed consent form showed the details of the study that were provided for the participants. Detailed information on age, gender and anthropometric measurements were collected through a face-to-face interview with the participants of the study.

### Anthropometric measurements 

Body weight and height were measured using a stadiometer (Seca, Germany), respectively barefoot and wearing light clothing to the nearest 0.1 kg for weight and 0.1 cm for height. WC and hip circumference (HC) were measured using an un-stretchable tape in a standing position without any pressure on the bodily surface, accurate to 0.1 cm. WC and HC were measured using the middle of the lowest gear, the high point of the iliac crest and on the biggest environmental gluteal muscle respectively. BMI was calculated by dividing weight/height^2^(kg/m^2^). WHR was estimated by WC (cm) divided by HC (cm) and WHtR as WC (cm) was divided by height (cm). BMI cut-off point for obese people in Asian subjects was selected for this population, according to WHO recommendation (BMI ≥25 kg/m^2^).^22^

### Statistical analysis

All data were analyzed using STAT software version14 (State Corp. College station, Texas, USA). Numeric and categorical variables were presented using mean (SD) and frequency (percent) respectively. To assess the diagnostic value of the predictors BMI, WC, WHR and WHtR we used receiver operating characteristic (ROC) curve. To achieve the optimal combination of sensitivity and specificity, we decided the best cut-off values of predictors for the outcome and then measured the positive predictive values, negative predictive values and positive and negative likelihood ratios (LR) along with their 95% CIs. Furthermore, the area under curve (AUC) and its 95% CI were presented as a measure ROC adequacy in diagnosing the outcome by the predictors. In addition the Hanley test to compare the ROC areas were used to identify the anthropometric indices which the best predicting the outcome.^23^ *P* values of less than 0.05 was regarded statistically significant.

## Results

The anthropometric characteristics of the study subjects have been shown in Table 1. The study sample was comprised of 215 women (43%) and 285 men (57%), with a mean age (SD) of 31.27 (10.53) and 40.33 (13.13) years respectively. In the present study, the number of participants with normal weight, overweight, and obesity was 173 (34.61%), 211 (42.19%), and 116 (23.20%), respectively.

According to Table 2, the mean of WC, WHtR and WHR was significantly different between the two groups of BMI (<25 and ≥ 25) in both women and men. Table 3 shows that the strongest association between the anthropometric measurements was found for WC and WHtR in both genders (r = 0.98) while the weakest correlation was observed for WC with WHR (r = 0.70) and WHtR in women (r = 0.71), respectively.

**Figure 1:**
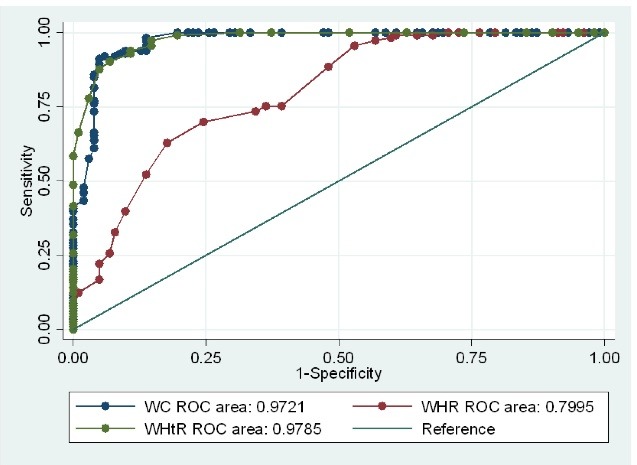
Comparison of AUC of the anthropometric indices for diagnosis of overweight and obesity in women. There were significant differences between three predictors in predicting overweight and obesity with meaningful higher AUC for WHtR (*P* < 0.05).

**Figure 2:**
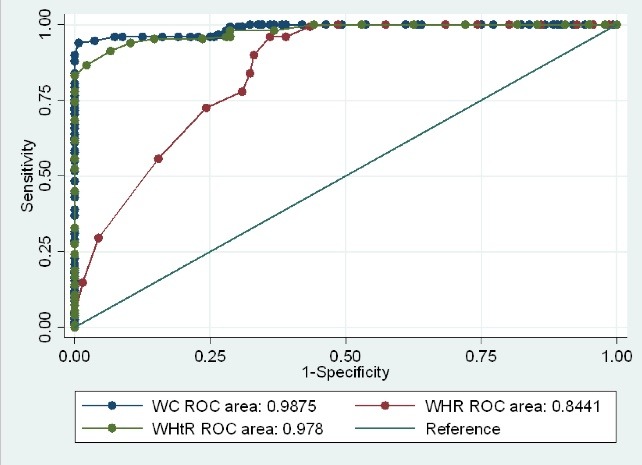
Comparison of AUC of the anthropometric indices for diagnosis of overweight and obesity in men. There were Significant differences between three predictors in predicting overweight and obesity with meaningful higher AUC for WC (*P* < 0.05).

The area under the ROC curve of WHtR (women: AUC = 0.97, 95%CI: 0.96-0.99vs. men: AUC = 0.97, 95% CI: 0.96-0.99) and WC (women: AUC = 0.97, 95% CI, 0.95-0.99 vs. men: AUC = 0.98, 95% CI: 0.97-0.99) were both greater than WHR (women: AUC = 0.79, 95% CI = 0.74-0.85 vs. men: AUC = 0.84, 95% CI = 0.79-0.88) (Figures 1 and 2).

WHtR and WHR demonstrated the highest sensitivity versus overweight and obesity in women (97.3%) and men (95.9%), respectively. Furthermore, WC revealed the highest specificity in women (94.1%) and men (99.2%) as well as the highest likelihood ratio in both women and men (15.6, 127.7 respectively). Sensitivity for WHtR showed that 97.3% of women and 91.2% of men are at risk of overweight and obesity (BMI ≥25 kg/m^2^) as they had WHtR values higher than 0.48 and 0.56 respectively. Specificity for WC, it revealed that 94.1% of healthy women (BMI <25 kg/m^2^) and 99.2% of healthy men had WC values lower than 84 cm and 98cm respectively. The LR+ (Positive Likelihood Ratio) for WC demonstrated that where the WC ≥84 cm, women are 15.6 times more likely to be at risk of overweight and obesity (BMI ≥25 kg/m^2^, true positive) than they were of not being at risk of overweight and obesity (BMI< 25 kg/m^2^, false positive). In addition, the LR+ for WC showed that in at WC ≥98, men are 127.7 times more likely to be at risk of being overweight and obese than not at risk. The LR- (negative likelihood ratio) for WC showed that the false negative rate was 0.08 and that it is 0.06 times more likely than the true negative rate in the WC <84 cm and WC <94 cm in women and men respectively (Tables 4 and 5).

## Discussion

The results of the present study indicated that, WC and WHtR were the best diagnostic indicators of obesity and overweight in comparison with WHR. According to our statistical analysis, the AUC of 1 demonstrates that the diagnostic test is perfect.^23^ Therefore, WC and WHtR, with an AUC of 0.972 and 0.978 in women and 0.987 and 0.978 in men, respectively, had a greater diagnostic ability to assess the status of being overweight and obesity than WHR with an AUC of 0.79 and 0.84 in men and women, respectively. Moy and Atiya compared the WC and WHR indicators with BMI among Malaysian men and women with age range between 20 and 58 years old.^14^ They found that WC used in obesity screening was stronger than WHR and that it could be an alternative index in terms of weight management apart from BMI. Studies conducted on German adult women and men as well as Australian women and men with age range between 20-69 years old also showed a strong relationship between WC and BMI and a weak correlation between WHR and BMI.^24,25^ Using computerized tumor (CT) scanning as a gold standard, a study focused on Brazilian adults of both sexes^26^ and women before menopause^27^ confirmed that, there is a strong relationship between WC and body fat and a weak relationship between WHR and body fat. In a study using DEXA technique, it was observed that the AUC for WC and BMI was high (0.76-0.92) in both sexes and that, the AUC was a little lower for WHR (0.74-0.88).^28^ In a study conducted by Bazhan et al on high school girls, there was a weak but significant relationship between WHR and BMI (r = 0.35) by considering a cut-off point of >0.8 for WHR.^29^ Unlike the above results, Esmaillzadeh et al introduced WHR as the most prominent indicator in predicting cardiovascular diseases compared to WC and WHtR. Several reasons may account for the discrepancy in findings.^30^ The predictive power of WC depends on population and varies from race to race. Ethnic and cultural diversity/differences are considered as a reason for the major conflict on the differences in the WC measurement. Differences in the BMI as well as age range of subjects studied can also lead to different findings. Generally, according to the results of the studies, WHR has little ability in case of diagnosis of being overweight and obesity compared to the two other indicators.^15,31^

The findings of this study showed that the AUC for WC and WHtR were similar and they did not differ significantly. The AUC for WHtR was slightly greater in women (0.978 versus 0.972), while the AUC for WC was slightly higher among men (0.987 versus 0.978). In the study by Heidari-Beni et al who investigated the diagnostic value of anthropometric indicators, both indices (WC and WHtR) had high sensitivity and WC had a higher LR+ compared to the other indices.^32^ In addition, a study conducted by Hsieh e al in Japan, demonstrated that WHtR acted better than the other indices when the metabolic risks in both sexes that were normal or overweight were identified.^33^ In Ho and colleagues’ study, which investigated anthropometric indices against CVD risk factors, WHtR had a higher sensitivity and lower LR+ compared to WC and WHR.^34^

The main problem with the use of the WHR indicator is that the size of the waist and hips usually varies greatly in the same way at the time of reduction or increase. Both WC and HC increase during weight gain, so we estimate that the effect of weight gain on this index will be less than the actual rate. Based on results of the studies, it seems that this index is not suitable for evaluating obesity, especially the processes of change related to weight. WHtR can be considered as an important index of body fat, because there are no significant changes in adult height. In addition, by measuring height, the weakness of the WC measurement can be reduced, as it can be used to eliminate height differences.^35^ WC is considered to be a good predictor for evaluating intra-abdominal fat (visceral fat) that is active metabolically.^36,37^ Some studies have indicated that this index can be used alone as a screening tool for overweight and obesity identification instead of BMI in weight management and control.^14,38^

In this study, we recommend the cut-off point of 84 cm for WC for women with a sensitivity and specificity of 92% and 94.1%, respectively, to determine overweight and obesity in the Iranian population. For men, we recommend the cut-off point of 98 cm for WC with a sensitivity and specificity of 93.9% and 99.2%, respectively in this population. In Western communities, the optimal cut-off points for WC are 102 cm for men and 88 cm for women.^39^ In a study by Heshmati et al, the suggested WC cutoff was 94.25 cm and 99.5 cm for women and men, respectively, to predict obesity in the Iranian adult population.^40^

Our study suggests a WHtR of 0.48 for women and 0.56 for men. WHtR has already been introduced as a common measure of central obesity for Asian societies and it has been suggested as a better indicator of CVD and mortality.^34,41^ The cut-off value of 0.5 for WHtR has been proposed for both sexes among European populations.^42^ In the present study, the WHR cut-off values used for diagnosing of being overweight and obesity were 0.78 and 0.87 for women and men, respectively, which both were less than the suggested cut-off points for European populations.

Study design was the main limitation of this study. Due to the cross-sectional nature of the study, causality cannot be deduced. Accordingly, future studies using longitudinal data will provide stronger evidence on this evaluation. Although estimating body weight percentage using bioelectrical impedance analysis (BIA) is the most reliable and valid technique for the assessment of obesity, unfortunately, the BIA technique was not available for the analyzes in our study, which could be considered as another limitation of the present study. Moreover, determination of WC, WHtR, or WHR optimal cut-off values for prediction of consequences/comorbidities associated with obesity should be considered in future studies.

## Conclusion

In this study, the WC and WHtR indicators were stronger indicators compared to the others. However, further studies using desirable and local cutoffs against more accurate techniques for body fat measurement such as CT scan and DEXA are required.

## Ethical approval

This study was approved by Ethics Committee of Tabriz University of medical sciences (IR.TBZMED.REC.1397.400). After being given a full explanation of the study procedures, the participants signed and informed consent form at baseline.

## Competing interests

The authors declared no potential conflicts of interest with respect to the research, authorship, and/or publication of this article.

## Funding

Nutrition Research Center of Tabriz University of Medical Sciences.

## Authors’ contributions

Author contributions were as follows: HT, AO, MEM, and MAJ designed the study and contributed to the conception of the project, development of overall research plan, and study oversight. HT drafted the manuscript and interpreted the data. MAJ contributed to the data analysis. All approved the final version of this manuscript.

## Availability of data and material

Data will be available from the corresponding author on reasonable request.

## Acknowledgments

The authors are grateful for the financial support of the Nutrition Research Center of Tabriz University of Medical Sciences. Also, we would like to thank all the patients who participated in this study.

**Table 1:** Anthropometric characteristics of subjects

	**Women (n= 215)**	**Men (n=285)**	***P*** **value**
	**Mean (SD)**	**Mean (SD)**	
Age (year)	31.27 (10.53)	40.33 (13.13)	<0.001
Weight (kg)	62.72 (15.51)	75.95 (18.55)	<0.001
Height (m)	159.2 (4.87)	174.77 (61.63)	<0.001
WC (cm)	82.68 (13.83)	97.82 (17.18)	<0.001
Hip circumference (cm)	101.47 (12.05)	109.36 (13.82)	<0.001
BMI (kg/m^2^)	24.66 (5.81)	24.95 (5.88)	0.582
WHtR	0.51 (0.08)	0.55 (0.10)	<0.001
WHR	0.80 (0.07)	0.88 (0.05)	<0.001

BMI, body mass index; WC, waist circumference; WHtR, waist-to-hip ratio; WHR, waist-to-height ratio.

**Table 2:** Mean and standard deviations of anthropometric indices through BMI categories

	**BMI <25 kg/m** ^ 2 ^		**BMI ≥25 kg/m** ^ 2 ^		***P*** **value**
	**Women**	**Men**	**Women**	**Men**	
	**Mean (SD)**	**Mean (SD)**	**Mean (SD)**	**Mean (SD)**	
WC	78.30 (9.83)	90.75 (13.01)	105.23 (8.40)	120.38 (5.22)	<0.001
WHtR	0.48 (0.06)	0.51 (0.76)	0.64 (0.05)	0.68 (0.04)	<0.001
WHR	0.79 (0.07)	0.86 (0.05)	0.87 (0.03)	0.93 (0.01)	<0.001

BMI, body mass index; WC, waist circumference; WHtR, waist-to-hip ratio; WHR, waist-to-height ratio.

**Table 3:** Pearson correlation coefficients between anthropometric indices

		**WC (cm)**	**WHtR**
Men	WC	1	
	WHtR	0.98^a^	1
	WHR	0.84^a^	0.83^a^
Women	WC	1	
	WHtR	0.98^a^	1
	WHR	0.70^a^	0.71^a^

WC, waist circumference; WHtR, waist-to-hip ratio; WHR, waist-to-height ratio. ^a^ *P* < 0.01.

**Table 4:** Area under the receiver operating characteristic (ROC) curve (95% confidence interval) and optimal cut-off values of diagnostic measures of overweight and obesity indices in women

	**AUC (95% CI)**	**Cut-off**	**Sensitivity %**	**Specificity %**	**LR+**	**LR-**
WC (cm)	0.97 (0.95-0.99)	84	92.0	94.1	15.6	0.08
WHtR	0.97 (0.96-0.99)	0.48	97.3	85.2	6.61	0.03
WHR	0.79 (0.74-0.85)	0.78	95.5	47.06	1.8	0.09

WC, waist circumference; WHtR, waist-to-hip ratio; WHR, waist-to-height ratio; LR, likelihood ratio; AUC, area under curve.

**Table 5:** Area under the receiver operating characteristic (ROC) curve (95% confidence interval) and optimal cut-off values of diagnostic measures of overweight and obesity indices in men

	**AUC (95% CI)**	**Cut-off**	**Sensitivity %**	**Specificity %**	**LR+**	**LR-**
WC (cm)	0.98 (0.97-0.99)	98	93.9	99.2	127.7	0.06
WHtR	0.97 (0.96-0.99)	0.56	91.2	93.3	13.79	0.09
WHR	0.84 (0.79-0.88)	0.87	95.9	63.9	2.66	0.06

WC, waist circumference; WHtR, waist-to-hip ratio; WHR, waist-to-height ratio; LR, likelihood ratio; AUC, area under curve.
